# Editorial: Spectroscopy for crop and product phenotyping

**DOI:** 10.3389/fpls.2022.1058333

**Published:** 2022-11-04

**Authors:** Ruslan Kalendar, Kioumars Ghamkhar, Pietro Franceschi, Marcos Egea-Cortines

**Affiliations:** ^1^ Helsinki Institute of Life Science HiLIFE, University of Helsinki, Biocenter 3, Helsinki, Finland; ^2^ National Laboratory Astana, Nazarbayev University, Astana, Kazakhstan; ^3^ Margot Forde Germplasm Centre, Grasslands Research Centre, AgResearch, Palmerston North, New Zealand; ^4^ Unit of Computational Biology, Research and Innovation Centre, Fondazione Edmund Mach, San Michele all’Adige, Italy; ^5^ Instituto de Biotecnología Vegegal, Universidad Politécnica de Cartagena, Cartagena, Spain

**Keywords:** spectroscopy, plant biochemistry, metabolites, Raman spectroscopy, near-infrared spectroscopy, fluorescence resonance energy transfer, hyperspectral imaging, genotyping

Spectroscopy and spectral imaging are suitable techniques for exploring plant biochemistry in an efficient, accurate and typically non-destructive manner (Schie et al., 2018). By taking advantage of the reflectance or absorbance properties of plant biomolecules and metabolites in different regions of the electromagnetic spectrum, today’s technologies allow for investigations into previously inaccessible aspects of biology in real-time (Akhgar et al., 2020). Other practical uses of the technology include biotic stress detection (early disease diagnostics), early abiotic stress detection, plant quality assessment or identification of species composition (Gemperline et al., 2016; Shorten et al., 2019; Li et al., 2020).

High-throughput technologies for nucleotide sequence analysis and detection of sequence variation have been increasingly used for plant genotyping and other fields of genetic testing. An important prospective use of PCR-based genotyping assays is to perform large-scale phenotyping analyses (Suyama and Matsuki, 2015), mutant screens, and comparative physiological analyses.

This Research Topic highlights novel and innovative applications of spectroscopic and spectrometric techniques, often coupled with advanced data analysis strategies, that aim for characterization in plants to understand plant traits with impact on growth and productivity. Studies featuring impactful and innovative applications of well-established methodologies such as Raman spectroscopy, Near-infrared spectroscopy, Fluorescence resonance energy transfer, hyperspectral imaging, or a novel combination of spectrometric measurement techniques and novel spectrometric techniques were invited, resulting in 8 published articles.

## Infrared and near-infrared spectroscopy (NIR) spectroscopy

As previously mentioned, NIR spectroscopy is a non-invasive technology that can be deployed for monitoring biomolecules with reflectance or absorbance within a given range of wavelengths (Gillie et al., 2000). Wyngaard et al., show that infrared spectroscopy is implemented for continuous monitoring of key metabolites in grapevine organs throughout the growing season. The observed spectral changes led to the classification of grapevine organs, providing individualized calibrations to compensate for the heterogeneity in grapevines, as well as developing more robust prediction models.

Armstrong et al., investigate the feasibility of single kernel NIR spectroscopy for rapid determination of protein, oil, and weight in intact single sorghum seeds, highlighting the use of this non-destructive and quick method for screening these traits in sorghum breeding and industry applications. In the work of Ejaz et al., biochemical components of sorghum were predicted for enhancing grain sorting efficiency for food, feed, and fuel, using Fourier-transform NIR spectroscopy.

## Raman spectroscopy-based plant pathology diagnostics

Raman spectroscopy (RS) is a label-free, non-invasive, non-destructive spectroscopic technique that is effective for studying the chemical structure of analyzed samples (Cialla-May et al., 2022). This technique has been widely used among biochemists, and has now found applications in agronomy, plant pathology and physiology for analysis of plant health status.

Changes in plant biochemistry can be probed by Raman spectroscopy, allowing its use in confirmatory diagnosis of plant pathology. Dou et al., use RS to develop the diagnosis of Huanglongbing, a devastating disease caused by *Candidatus Liberibacter* spp. (Ca. L. asiaticus). By using a combination of HPLC and image studies of leaves, they created a ground truth concept demonstrating that a given signature in RS corresponds to increased p-coumaric acid and decreased lutein in infected grapefruit leaves. Since Raman spectroscopy can be used to resolve stress-induced changes in plant biochemistry on the molecular level, it represents a prospective and rapid technique for agronomy and plant pathology. Farber et al., show that RS can be used for highly accurate identification of stalk rot caused by *Colletotrichum graminicola* in maize at both early and late stages of disease progression, *via* spectroscopic analysis of both leaves and stalks.

## High-resolution microscopy and mass spectrometric imaging

The rhizosphere is a hotspot for microbial activity, organic carbon input, and carbon turnover in soils (Ilhardt et al., 2019). Several stand-alone and combinatorial methods have been developed to investigate the chemistry and the role of microbes in soil and the rhizosphere.

Bandara et al., present a novel approach that allows simultaneous microbial identification and chemical analysis of the rhizosphere at a spatial resolution ranging from micro- to nanometers. This new method allows for comprehensive study of the spatio-temporal organization of nutrients and microbes in the rhizosphere at an unprecedented scale and provides a platform for a mechanistic understanding of complex patterns of interactions between roots, the microbiome and soil using a correlative microscopy approach. Lohse et al., present a novel workflow using laser desorption ionization combined with mass spectrometric imaging to directly analyze plant metabolites in a complex soil matrix. The target metabolites were detected with a spatial resolution of 25 μm in the root and surrounding soil, based on accurate masses using ultra-high mass resolution laser desorption ionization Fourier-transform ion cyclotron resonance mass spectrometry. Direct molecular imaging allows a non-targeted or targeted analysis of plant metabolites in undisturbed soil samples, paving the way to study the turnover of root-derived organic carbon in the rhizosphere with high chemical and spatial resolution.

## Fluorescence resonance energy transfer-based genotyping

Single-nucleotide polymorphisms (SNPs) represent the smallest type of genetic differences in DNA between biological samples (Campbell et al., 2015). Fluorescence resonance energy transfer is a popular detection method for SNP analysis and genotyping based on distinctly different platforms and approaches. Kalendar et al., propose a modification to improve the version of the existing Allele-specific PCR method that is similar to the Kompetitive allele specific PCR (KASP) technique (LGC Biosearch Technologies) for genotyping SNPs based on fluorescence resonance energy transfer. This new technique is based on the simultaneous presence of two components in the PCR: an allele-specific mixture (allele-specific and common primers), and a template-independent detector mixture that contains two to four universal probes and a single universal quencher oligonucleotide (Kalendar et al., 2022). The proposed method was used for SNP genotyping in barley genes HvSAP16 and HvSAP8, and is suitable for bi-allelic uniplex, 3- or 4-allelic variants, or different SNPs in a multiplex format that can be used in a range of applications including medical, forensic, or any study involving SNP genotyping.

Overall, the research collected on this Research Topic highlights innovative and promising applications of all spectroscopic techniques for characterizing plants to understand plant growth, productivity, and disease resistance, and for PCR-based genotyping to perform large-scale mutant screens.

## Author contributions

RK prepared the draft. All authors listed have made a substantial, direct, and intellectual contribution to the work and have approved it for publication.

## Funding

This work was supported by the Science Committee of the Ministry of Education and Science of the Republic of Kazakhstan (OR11465422) to RK and by New Zealand’s Ministry of Business, Innovation and Employment to KG.

## Acknowledgments

We thank all authors and reviewers for their contributions to this Research Topic and for the support of the editorial office.

## Conflict of interest

The authors declare that the research was conducted in the absence of any commercial or financial relationships that could be construed as a potential conflict of interest.

## Publisher’s note

All claims expressed in this article are solely those of the authors and do not necessarily represent those of their affiliated organizations, or those of the publisher, the editors and the reviewers. Any product that may be evaluated in this article, or claim that may be made by its manufacturer, is not guaranteed or endorsed by the publisher.
